# Supplementary material for: A fault-intrusion-tolerant system and deadline-aware algorithm for scheduling scientific workflow in the cloud
Source: PeerJ Comput Sci. 2021 Nov 2;7:e747. doi: 10.7717/peerj-cs.747 (PMC8576554; doi:10.7717/peerj-cs.747)
Supplement: Supplemental Information 6 [file peerj-cs-07-747-s006.zip › Myproject1.1/docs/org/workflowsim/utils/ReplicaCatalog.html]

ReplicaCatalog


JavaScript is disabled on your browser.


- Overview
- Package
- Class
- Use
- Tree
- Deprecated
- Index
- Help

- Prev Class
- Next Class

- Frames
- No Frames

- All Classes

- Summary:
- Nested |
- Field |
- Constr |
- Method

- Detail:
- Field |
- Constr |
- Method


org.workflowsim.utils

## Class ReplicaCatalog

- java.lang.Object
- - org.workflowsim.utils.ReplicaCatalog

- ---

    

  ```
  public class ReplicaCatalog
  extends java.lang.Object
  ```

  ReplicaCatalog stores all the data information and where (site) there are

  Since:
  :   WorkflowSim Toolkit 1.0

- - ### Nested Class Summary

    Nested Classes

    | Modifier and Type | Class and Description |
    | --- | --- |
    | `static class` | `ReplicaCatalog.FileSystem` File System |
  - ### Constructor Summary

    Constructors

    Constructor and Description

    `ReplicaCatalog()`
  - ### Method Summary

    Methods

    | Modifier and Type | Method and Description |
    | --- | --- |
    | `static void` | `addStorageList(java.lang.String file, java.lang.String storage)` Adds a file to a storage |
    | `static boolean` | `containsFile(java.lang.String fileName)` Checks whether a file exists |
    | `static File` | `getFile(java.lang.String fileName)` Gets the file object based its file name |
    | `static ReplicaCatalog.FileSystem` | `getFileSystem()` Gets the file system |
    | `static java.util.List` | `getStorageList(java.lang.String file)` Gets the list of storages a file exists |
    | `static void` | `init(ReplicaCatalog.FileSystem fs)` Initialize a ReplicaCatalog |
    | `static void` | `setFile(java.lang.String fileName, File file)` Adds a file name and the associated file object |

    - ### Methods inherited from class java.lang.Object

      `clone, equals, finalize, getClass, hashCode, notify, notifyAll, toString, wait, wait, wait`

- - ### Constructor Detail


    - #### ReplicaCatalog

      ```
      public ReplicaCatalog()
      ```
  - ### Method Detail


    - #### init

      ```
      public static void init(ReplicaCatalog.FileSystem fs)
      ```

      Initialize a ReplicaCatalog

      Parameters:
      :   `fs` - the type of file system


    - #### getFileSystem

      ```
      public static ReplicaCatalog.FileSystem getFileSystem()
      ```

      Gets the file system

      Returns:
      :   file system


    - #### getFile

      ```
      public static File getFile(java.lang.String fileName)
      ```

      Gets the file object based its file name

      Parameters:
      :   `fileName,` - file name

      Returns:
      :   file object


    - #### setFile

      ```
      public static void setFile(java.lang.String fileName,
                 File file)
      ```

      Adds a file name and the associated file object

      Parameters:
      :   `fileName,` - the file name
      :   `file` - , the file object


    - #### containsFile

      ```
      public static boolean containsFile(java.lang.String fileName)
      ```

      Checks whether a file exists

      Parameters:
      :   `fileName` - file name

      Returns:
      :   boolean, whether the file exist


    - #### getStorageList

      ```
      public static java.util.List getStorageList(java.lang.String file)
      ```

      Gets the list of storages a file exists

      Parameters:
      :   `file` - the file object

      Returns:
      :   list of storages


    - #### addStorageList

      ```
      public static void addStorageList(java.lang.String file,
                        java.lang.String storage)
      ```

      Adds a file to a storage

      Parameters:
      :   `file,` - a file object
      :   `storage` - , the storage associated with this file


- Overview
- Package
- Class
- Use
- Tree
- Deprecated
- Index
- Help

- Prev Class
- Next Class

- Frames
- No Frames

- All Classes

- Summary:
- Nested |
- Field |
- Constr |
- Method

- Detail:
- Field |
- Constr |
- Method
